# Gut bacterial population and community dynamics following adult emergence in pest tephritid fruit flies

**DOI:** 10.1038/s41598-023-40562-2

**Published:** 2023-08-22

**Authors:** Charles J. Mason, Jean Auth, Scott M. Geib

**Affiliations:** Tropical Pest Genetics and Molecular Biology Research Unit, Daniel K Inouye U.S. Pacific Basin Agricultural Research Center, Agricultural Research Service, USDA, 64 Nowelo Street, Hilo, HI 96720 USA

**Keywords:** Microbial ecology, Microbiome, Entomology

## Abstract

Gut microbiota are important contributors to insect success. Host-microbe interactions are dynamic and can change as hosts age and/or encounter different environments. A turning point in these relationships the transition from immature to adult life stages, particularly for holometabolous insects where there is radical restructuring of the gut. Improved knowledge of population and community dynamics of gut microbiomes upon adult emergence inform drivers of community assembly and physiological aspects of host-microbe interactions. Here, we evaluated the bacterial communities of the pest tephritid species melon fly (*Zeugodacus cucurbitae*) and Medditeranean fruit fly (medfly, *Ceratitis capitata*) associated with the pupae life stage and timepoints immediately following adult eclosion. We used a combination of culturing to determine cultivatable bacterial titers, qPCR to determine 16S-rRNA SSU copy numbers, and 16S V4 sequencing to determine changes in communities. Both culturing and qPCR revealed that fly bacterial populations declined upon adult emergence by 10 to 100-fold followed by recovery within 24 h following eclosion. Titers reached ~ 10^7^ CFUs (~ 10^8^ 16S rRNA copies) within a week post-emergence. We also observed concurrent changes in amplicon sequence variance (ASVs), where the ASV composition differed overtime for both melon fly and medfly adults at different timepoints. Medfly, in particular, had different microbiome compositions at each timepoint, indicating greater levels of variation before stabilization. These results demonstrate that tephritid microbiomes experience a period of flux following adult emergence, where both biomass and the makeup of the community undergoes dramatic shifts. The host-microbe dynamics we document suggest plasticity in the community and that there may be specific periods where the tephritid gut microbiome may be pliable to introduce and establish new microbial strains in the host.

## Introduction

Insects form intimate associations with microorganisms in their digestive systems, which collectively can contribute to important physiological and ecological functions^1,2^. Host-microbe interactions are not necessary static, and microbiome compositions fluctuate within individuals under different dietary conditions, developmental stage, and time. This is especially the case for insects undergoing complete metamorphosis (holometabolism). A combination of gut morphological and biochemical alterations, host dietary selection and utilization, as well as the different needs and longevity of the life stages can lead to altered microbial relationships^3^. Indeed, the dynamic and divergent nature of gut microbiomes between immatures and adults have been demonstrated for members of the Hymenoptera, Coleoptera, Lepidoptera, and Diptera orders^4–9^.

Determining bacterial population and community dynamics of gut microbiomes can provide insight into how hosts regulate symbiont assemblages. Furthermore, fine-scale evaluation of microbiome population dynamics following significant life events allows exploration into how host-microbe dynamics recover from physiological and environmental perturbations. Our principal aim was to elucidate the gut microbiome population and community dynamics in insects following adult emergence, and whether patterns of establishment vary between different species and from different larval diets. Specifically, we evaluated gut bacteria of two important pest species of true fruit flies (Diptera: Tephritidae).

Tephritid fruit flies are globally distributed pests of ripening fruit and can be a significant impediment to agriculture and trade^10–12^. Tephritids are known to harbor a diverse assemblage of microorganisms in their gut tissues which include can fungi and bacteria^13–15^. Bacteria can be important components of both the tephritid larval and adult gut microbiome, having roles in facilitating nutrient provisioning and improving fitness^16–18^. As is the case with many holometabolous insects, tephritids consume divergent diets as adults and larvae, and their gut microbiomes can reflect these substantial changes^6,19^. Understanding the establishment and flexibility of gut microbiome dynamics would help inform sources of variation that are often observed in natural populations of males^20^ as well as how host-microbe respond to different environments^21^. Additionally, while determining microbial dynamics of flies in natural settings can uncover fundamental physiological functioning, elucidating how microbial establishment occurs in insectary-maintained populations is especially important for tephritid fruit flies. Establishment of pest tephritids into novel agricultural areas is often mitigated through a combination of tactics targeting adults, including the sterile insect technique (SIT) for mating disruption^22,23^. Manipulation of microbiomes have been proposed for species reared for SIT^14,24,25^ and evaluating developmental windows to efficiently introduce and determine community resilience are still needed.

In this study, we assessed the quantitative and compositional changes in gut microbiota of two pest tephritid species following adult eclosion, with one species originating from two different larval diets. We evaluated the microbial populations and communities of the Mediterranean fruit fly (medfly, *Ceratitis capitata*) and melon fly (*Zeugodaucus cucurbitae*) from colony sources, and collected melon fly pupae from infested papaya fruit. We performed a series of culture- and DNA-based microbiota quantitation as well as 16S-rRNA SSU microbiome sequencing at multiple timepoints within three weeks following adult eclosion.

## Results

### Bacterial titers vary over short windows

**Figure 1:**
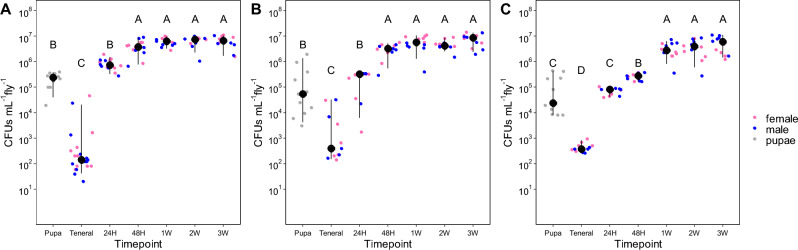
Colony forming units (CFUs) of melon fly originating from papaya (**A**) and colony sources (**B**) and medfly from a colony source (**C**) at different intervals. Black dots represent median with bars representing 95% confidence intervals. Letters represent significant differences between sampling timepoints (p < 0.05) using log_10_ transformed values.

In all fly species, there were significant changes in culturable microbial titers across developmental windows (Fig. 1; melon lab F_6,68_ = 128.1, p < 0.001; melon papaya F_6,83_ = 208.5, p < 0.001; medfly lab F_6,68_ = 123.1, p < 0.001). The most dramatic changes involved newly emerged (teneral) flies. CFUs sharply declined by 10–1000× upon adult emergence from pupae, followed by rapid increases (~ 300×) over a 24-h period. For both papaya and laboratory sourced melon fly (Fig. 1A,B), CFUs stabilized by 48 h following adult eclosion at ~ 4 × 10^6^ CFUs mL^−1^ per fly for remaining sampling time. Medfly increases after emergence were more gradual, increasing from 500 CFUs to ~ 6 × 10^4^ after 24 h, to ~ 3 × 10^5^ after 48 h, before stabilizing between 3–5 × 10^6^ after one week. We observed no indication that sex influenced CFU titers (melon lab F_1,68_ = 0.28, p = 0.599; melon papaya F_1,83_ = 1.27, p = 0.263; medfly lab F_1,68_ = 0.67, p = 0.414).

**Figure 2:**
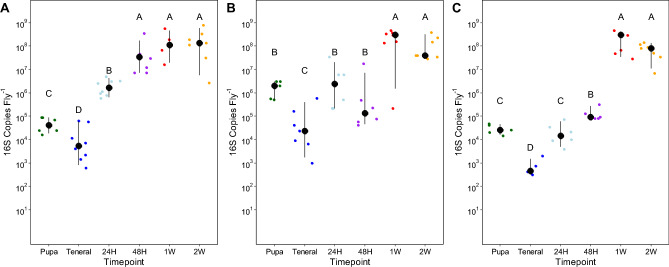
16S-rRNA copy numbers in extracted samples for melon fly reared from papaya (**A**) and obtained from colony (**B**) and medfly from colony sources (**C**). Black dots represent mean with bars representing 95% confidence intervals. Letters represent significant differences between sampling timepoints (p < 0.05) using log_10_ transformed values.

16S SSU rRNA copy numbers corroborated trends observed with culture-based approaches (Fig. 2; melon lab F_5,33_ = 83.4, p < 0.001; melon papaya F_5,35_ = 66.8, p < 0.001; medfly lab F_5,31_ = 109.9, p < 0.001). All species exhibited precipitous declines in 16S copy numbers upon adult eclosion from the pupal stages, followed by increases of 10–100× within 24 h. Papaya melon fly copies increased from ~ 10^4^ (per fly) to ~ 10^8^ within 48 h of eclosion (Fig. 2A), while laboratory melon fly copy numbers first increased from ~ 10^4^ to ~ 10^6^ after 48 h before stabilizing at 10^8^ after one week (Fig. 2B). 16S titers of freshly eclosed medfly increased from 10^3^ to 10^5^ copy numbers within 48 h before increasing to 10^8^ after one week (Fig. 2C).

### Fluctuations in fly 16S ASV composition

**Figure 3:**
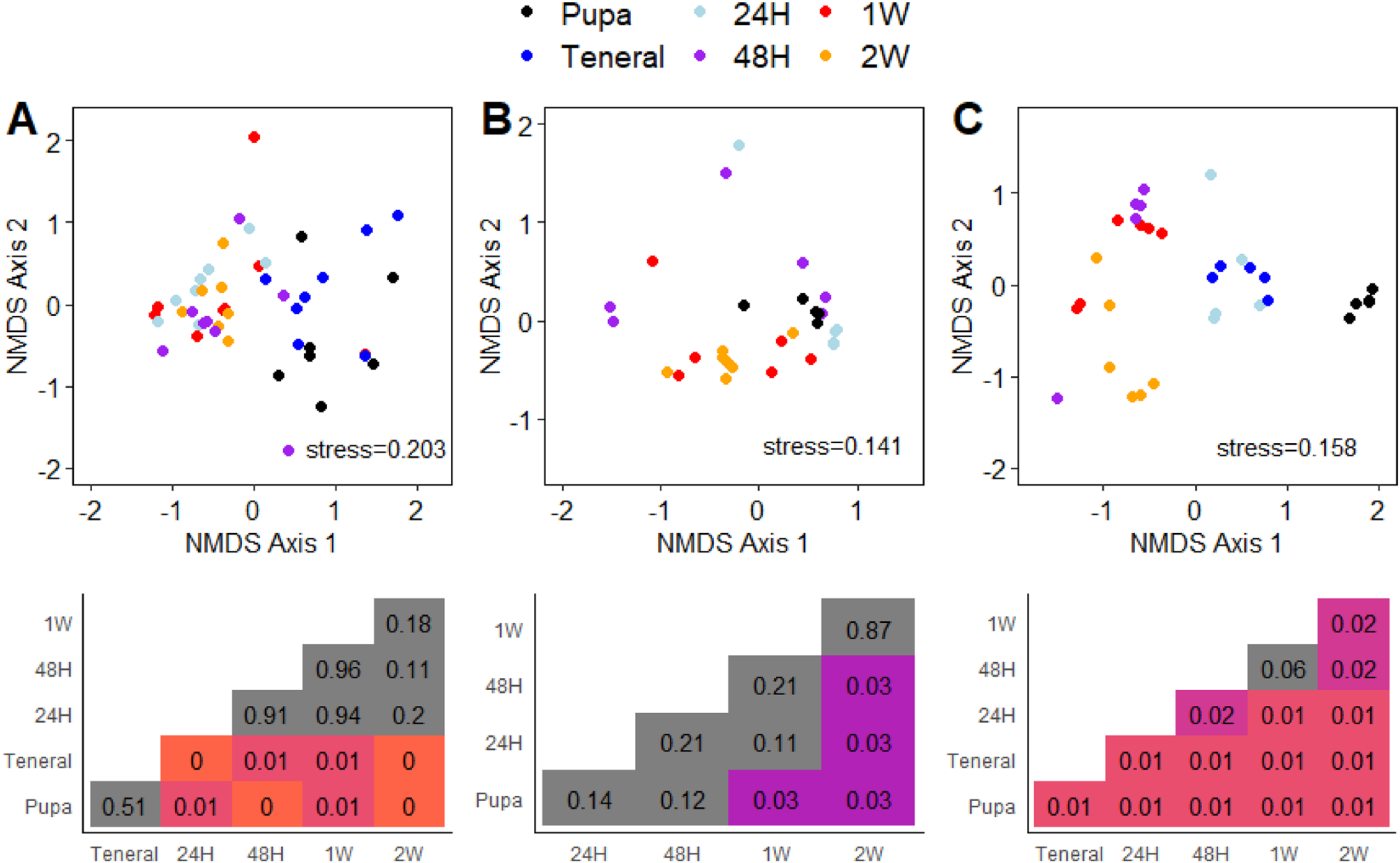
Non-metric multidimensional scaling (NMDS) plots and p-values of pairwise comparisons of V4 16S SSU rRNA ASVs using Bray–Curtis distances. Sample grouping include melon fly from papaya (**A**), melon fly from insectary (**B**), and medfly from insectary (**C**). Heatmaps illustrate pairwise comparisons between sample groups corresponding to NMDS plot immediately above the heatmap, where incidences where p > 0.05 are shaded gray.

**Figure 4:**
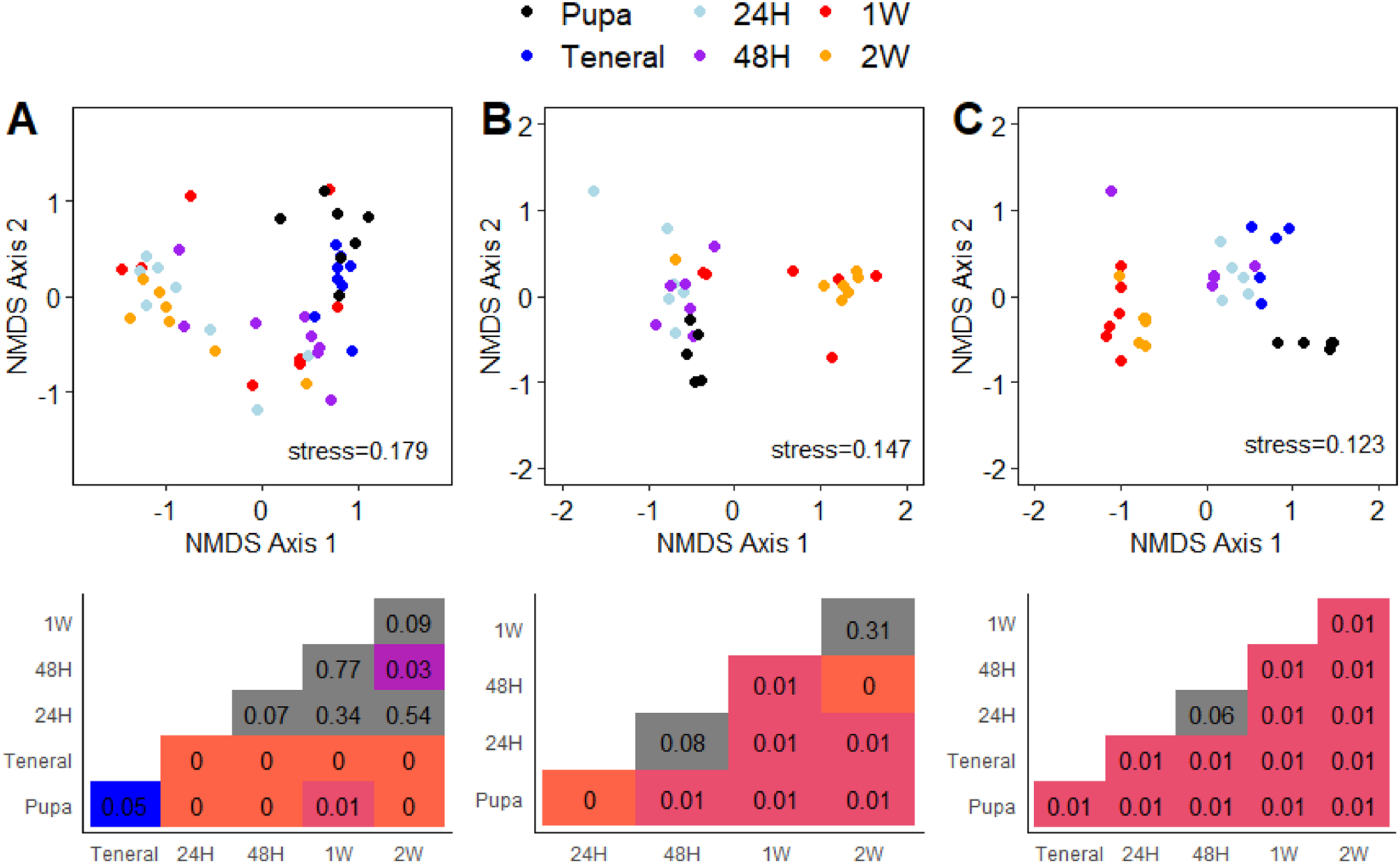
Non-metric multidimensional scaling (NMDS) plots and p-values of pairwise comparisons of V4 16S SSU rRNA ASVs using Jaccard distances on presence/absence data. Sample grouping include melon fly from papaya (**A**), melon fly from insectary (**B**), and medfly from insectary (**C**). Heatmaps illustrate pairwise comparisons between sample groups corresponding to NMDS plot immediately above the heatmap, where incidences where p > 0.05 are shaded gray.

While there were some effects of developmental timepoint on 16S rRNA ASV alpha-diversity metrics, the trends were generally inconsistent, and variable (Supplemental Table 1). These patterns contrasted with beta-diversity metrics in both ASV compositional (Bray–Curtis distances on relative abundances) and membership (Jaccard distances on presence/absence) for all groups of samples (Figs. 3 and 4, respectively). Nonetheless, there are some nuanced aspects of how the different fly species responded.

For melon fly from papaya, there was initially a high level of overlap in the composition of the communities between pupae and teneral adults (Fig. 3A). After 24 h, there was divergence in the communities of all subsequent samples from the pupal and teneral timepoints (F_df_^5,45 ^= 2.41 p < 0.001). While diverging from the earlier timepoints, the remaining timepoints had a high degree of overlap with each other and were not distinguishable (pairwise PERMANOVA p > 0.05). Presence/absence of ASVs largely followed the same trends (Fig. 4A) with some caveats; pupae and teneral adults were significantly different from all other timepoints and we also observed differences between the 48H timepoint and the 2W timepoint.

Melon fly pupae obtained from the PBARC insectary had a slightly different trajectory in composition than those from fruit based on pairwise PERMANOVA. ASV compositions associated with pupae began to differ between 1 and 2W, but not between other timepoints (Fig. 3B). Similarly, one week old flies did not differ from flies collected at 24H, 48H, or two weeks, suggesting a more gradual shift in the community. Analysis using presence/absence and Jaccard dissimilarities followed similar patterns (Fig. 4B). We recovered sequences for only a single teneral melon fly lab sample, so this group of samples were excluded from the analyses.

Microbiome composition and membership of medfly (Figs. 3C and 4C) varied across adult development. For medfly, all sample groups exhibited significantly different compositions with exception of between 48 h- and one-week old individuals. Jaccard dissimilarities (Fig. 4C) differed between each timepoint.

Since we had samples collected at the same timepoints for the individual species, we compared their respective ASV compositions at the pupal, 24 h, one-week, and two-week sampling points. For both beta-diversity metrics (Bray–Curtis and Jaccard dissimilarities), we observed significant differences between the fly species at each timepoint (Supplemental Fig. 1), indicating a host-specific signal of ASV composition.

### Taxonomic responses over time

**Figure 5:**
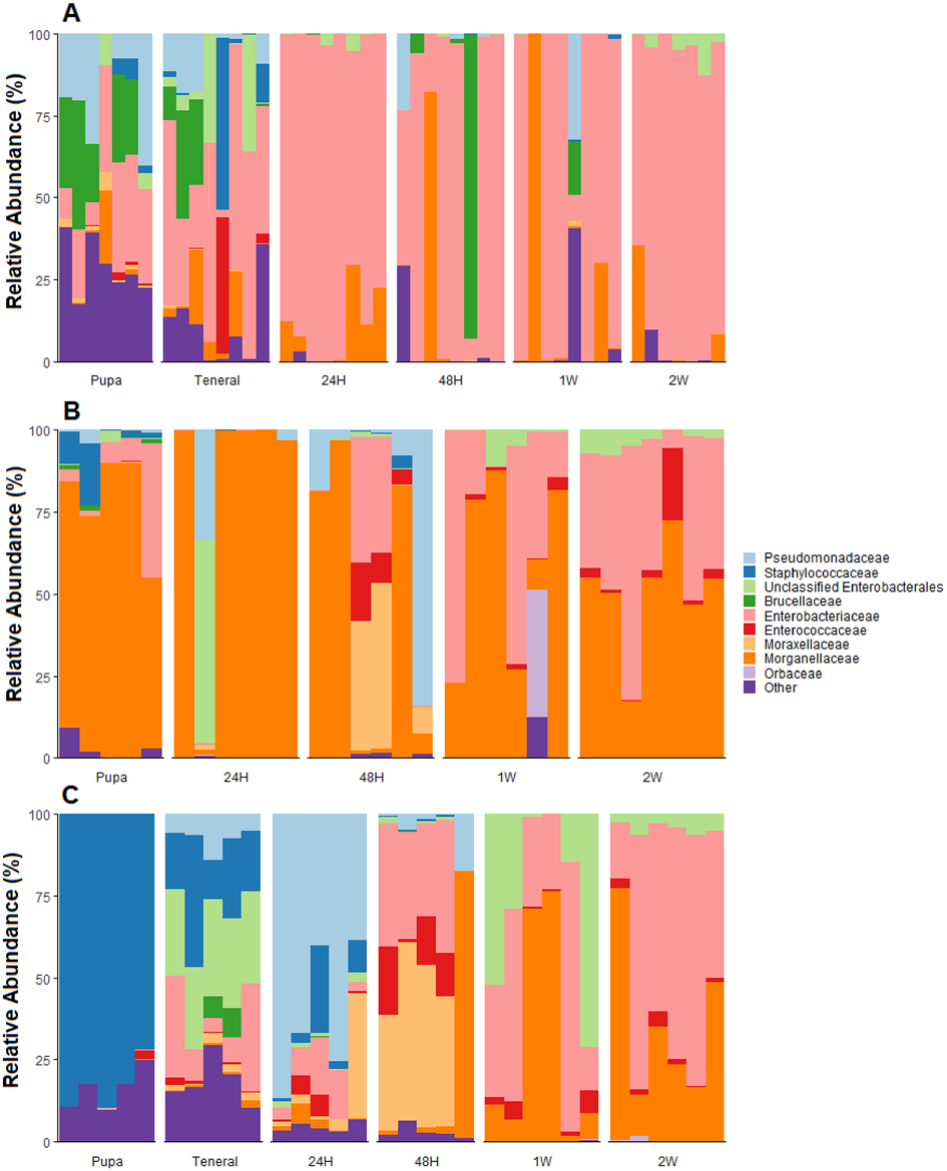
Relative abundances of 16S-rRNA reads at the family level associated with gut tissues from melon fly reared from papaya (**A**) and obtained from colony (**B**), and medfly from colony sources (**C**). ‘Other’ represents sequences represented in averaging less than 2% relative abundance across all samples. Taxonomies determined via an RDP dataset formatted for the DECIPHER package. If taxa were unclassified at the genus level, they were listed as unclassified at the next highest taxonomy.

**Figure 6:**
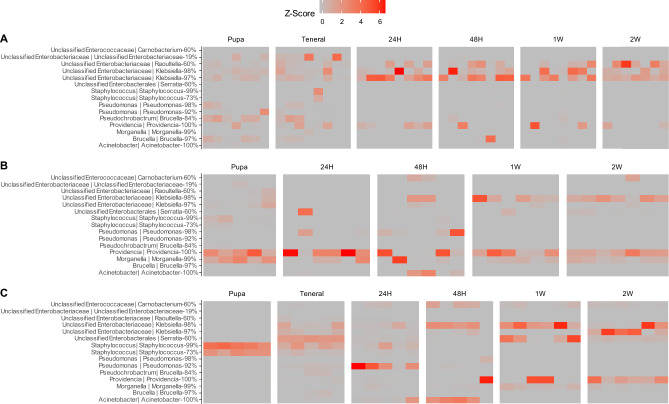
Heatmaps of individual ASVs through development of melon fly reared from papaya (**A**) and obtained from colony (**B**), and medfly from colony sources (**C**). Individual plots are provided in supplementary materials (Supplemental Figs. 3–5). Both taxonomies determined from DECIPHER and RDP are included (left, and right, respectively), with the RDP bootstrap value at the genus level included.

There were taxonomic shifts in 16S reads between the sample timepoints for all species surveyed. The magnitude of these changes varied between individual species (Figs. 5 and 6), and there were some patterns that appeared to be consistent among the four groups of specimens. In general, there was an increase in the relative abundances of the bacterial class Enterobacterales as fly aged (Fig. 5), with increases in Enterobacteriaceae and Moraxallaceae being among the most prevalent.

For melon fly from papaya, associations with specific taxa were more diffuse with the pupal stages (Fig. 5A), with only a handful of ASVs becoming most prevalent in the later stages. Specifically, relative abundances of ASVs corresponding to *Raoultella* and *Klebsiella* became most prevalent at 24 h-post eclosion and remained at the highest relative abundance for the other timepoints (Fig. 6A). However, none of these ASVs were significantly different across life stages (Supplemental Table 2), as they were present in pupae and teneral adults. Compared to the other species, the individual taxonomic responses were limited to decreases in ASVs at the pupal stages, following the trends supported in the beta-diversity measurements.

The laboratory melon fly responses were due to decreases in Morganellaceae in the pupal stages, and increases in the Enterobacteriaceae (Fig. 5B), but some ASVs were present in all stages. For instance, ASVs corresponding to *Providencia* and *Morganella* were observed in pupal stage and subsequently, with several *Providencia* ASVs increasing in relative abundance over time (Supplemental Table 3). Notably, two *Klebsiella* ASVs increased through time (Fig. 6B; Supplemental Table 3), where one was at low abundances in pupal stages (< 3%) and increasing to 25% of the relative abundance after one-week post-eclosion.

Laboratory medfly exhibited a stark amount of microbial turnover following eclosion. At the pupal stage, samples were dominated by ASVs belonging to the Staphylococcaceae (Fig. 5C; Supplemental Table 4). Upon eclosion, medfly samples underwent several transitions, first being populated by a diffuse assemblage as tenerals, before undergoing shifts in ASVs classified as *Pseudomonas*, *Morganella,* and *Acinetobacter* between 24 and 48 h post eclosion to being *Klebsiella* and *Providencia* as features after the two-week interval (Fig. 6C).

## Discussion

The transition from immature to adult for holometabolic insects involves substantial morphological, physiological, and life history changes^26^. Accordingly, changes in gut microbiomes also accompany the dramatic anatomical remodeling and life history changes that accompany metamorphosis^3,27^. Here, we demonstrate that gut bacterial titers and the bacterial compositions shift following the transition of pupa to adult in melon and medfly. Both species underwent consistent, abrupt changes in gut bacterial titers following adult eclosion, where there was a depletion in microbial biomass followed by rapid recovery. Additionally, we observed changes in microbiome composition over the two weeks following emergence. Each species harbored unique communities, suggesting host-specific selection of their respective gut microbiomes. Our results indicate there is a period of flux in the tephritid gut microbiome at the point of adult eclosion which may be exploited to introduce novel microbes to mass-reared insects.

Upon emergence, microbial biomass decreased for melon fly and medfly, including adults emerged from melon fly pupae collected from natural populations (Figs. 1 and 2). Microbial titers recovered within 48 h, increasing by > 100×. These patterns post-eclosion have been noted in microscopy-based studies of medfly adults^28^ and in amplicon sequencing efforts in the Queensland fruit fly (*Batrocera tyroni*)^29^. Comparable patterns—rapid alteration of microbial population dynamics during metamorphosis—have been documented for other insect species^7,30–32^, including flies^33–35^. While we did not study the larval transition here, some species purge their digestive systems of microbiota prior to pupation in addition to further depletion as emerging adults. This is appears to be the case for other dipterans such as *Drosophila*^35–37^, where there are sharp declines in titers upon emergence followed by a period of rapid recovery. For *Drosophila*, the larval gut is not fully histolyzed during pupation; instead the adult gut is constructed around the larval gut as a transient pupal epithelial layer^38^. After emergence, *Drosophila* excretes the remaining portions of the larval gut from the newly formed adult midgut^36^. While the exact mechanism that leads to precipitous decline of microbiota in tephritids is unknown, we can surmise that it is a combination of excretion upon adult eclosion and possible regulation of gut immunity^39,40^.

Accompanying fluctuations in microbial populations, we observed shifts in gut ASV composition in the days and weeks following adult eclosion. These data corroborate previous studies which surveyed microbial communities in tephritid fruit fly adults and immature stages over coarse timeframes^6,41–45^, including the species we surveyed here^19,46^. We expected a linkage between the initially decreased titers accompanying these compositional changes, as established gut microbiomes can restrict the colonization of other microorganisms^47,48^. Decreased titers may afford reconfiguration of the gut microbiome through microbe-microbe competition and within host propagation, ingestion of environmental organisms through foods^49,50^, and swapping of microbes through trophallaxis behaviors that occur in the species we investigated^51^. A “soft” reset of the gut microbiome through depletion may partially explain why tephritid fruit flies have been documented to exhibit high variability in the field and locality-specific effects^20,52^. Additionally, reconfiguration of gut microbiomes between adult and immature life stages may afford larvae to access a larger diversity of resources, with adults being populated by bacteria fulfilling core functions^6^.

Both melon and medfly remodeled their gut microbiomes following adult eclosion, with medfly exhibiting more dramatic changes. Such dynamics have been observed previously with medfly, where there are ontogenetic mediated changes through development and fluctuation of dominant microbiota^52^, resulting in fluctuation and reconfiguration between larvae and maturation of adults. Medfly pupae that were once dominated by *Staphylococcus* yielded adults possessing high abundances of Enterobacteriaceae (Fig. 5C; Supplemental Fig. 3) while melon fly pupae possessed a diverse set of taxa that were present later in development. The medfly larval diet is highly acidified for sanitation purposes in mass rearing^53^, while the PBARC melon fly lab diet is not (Supplemental Table 5). These results suggest that medfly might undergo some degree of environmental microbial acquisition or the Enterobacteriaceae are diminished in immature stages, and early periods of adult life being variable before stabilization of host microbiome composition. The contrast between medfly and the melon fly laboratory sources appear to support this notion; melon fly microbiome composition stabilized earlier than medfly following eclosion (Fig. 3) while also exhibiting a greater number of ASVs shared between adults and pupae (Supplemental Figs. 4 and 5). Representatives of the Enterobacteriaceae have been documented in wild and captive medfly microbiomes globally^29,54–58^. Members of the Enterobacteriaceae, like *Klebsiella* and *Enterobacter*, are likely physiologically important components of the adult medfly gut microbiome^14^. These observations raise questions about how microbial strains may vary among and between rearing populations, how priority effects influence the composition of fly microbiomes over longer durations, and how tephritid microbiome establishes during when the insect is under laboratory culture.

*Klebsiella* and other Enterobacteriaceae are often detected in sequencing and culturing efforts of tephritid fruit fly pests^21,41,46,56,57,59,60^, but how they are retained between lifestages, fluctuate in abundance relative to other microbial taxa, exhibit taxonomic and functional resiliency, and vary between insect populations and across timepoints are not well-resolved. Specifically, the amount of variation in specific isolates and strains, and how that might impact fly performance is unknown. Given its status as a global pest and the fact that there are apparent compositional differences in the medfly microbiome^58,61^, this is a major deficiency in our understanding of strain diversity in this system. For instance, our results and others^52^ indicate some reconfiguration of some degree across host development, but we do not know if new strains are entering the system, or whether it is the same strain that was once suppressed is reestablishing dominance. Utilizing other sequencing techniques (whole genome sequencing of isolates, shotgun metagenomics, or long read 16S) will help uncover these processes and allow for a more comprehensive understanding of strain dynamics.

Multiple studies over the past decade have shown that mass reared medfly for sterile insect technique (SIT) can harbor gut microbiomes that differ from the wild, and this may adversely impact male competitiveness^16,17,19,54^. Manipulating microbial assemblages of sterile medfly has been documented to improve fly competitiveness^17,18^, generating interest in potential application of microbiota for SIT programs^14,22^. Although application to SIT was outside the immediate scope of this study, our results indicate that there may be an opportunity to reintroduce wild bacterial strains to newly emerging flies from the laboratory.

As a part of our study, we collected infested pupae from wild melon fly populations to compare with those in a laboratory population. We observed consistent trends in microbial population dynamics between these two sources (16S copies and CFUs titers) as flies aged. Unexpectedly, the microbiomes differed between the two sources (Supplemental Fig. 1). While we observed differences ASV compositions, the fact that population responses were consistent between the sources suggests robustness in the post-eclosion population dynamics and reorganization in melon fly. However, this is a limited comparison and requires further exploration to determine the influence of host genetics and larval diet that may drive microbial community make-up.

Questions that arise from our observations involve the timeframes where the tephritid hosts control gut microbial colonization. Typically, a combination of antimicrobial peptides and reactive oxygen species are involved in the control and proliferation of microbial populations in the digestive tract^62–65^. We presume there is an upregulation of gut immune defenses to abate proliferation of gut microorganisms following adult eclosion, but speculation on exact mechanisms is tenuous. Deciphering host immune and other digestive responses in the period of microbial proliferation is important to understand host control of potential symbionts. Similarly, it would be important to know if the host is vulnerable to microbial invasion during this window, and uncovering host-mediated responses would inform aspects of community assembly. Further investigation in host-microbe dynamics after maturity as flies age is also needed. Specifically, for the tephritid systems we have relatively limited understanding regarding how host background interacts with gut microbiota to influence adult lifespan and fitness^66,67^.

Our results demonstrate that tephritid fruit flies undergo a period of flux of their microbial populations and communities following adult eclosion. These data confirm trends observed elsewhere for tephritid fruit flies^28,29^, and provide insights into bacterial colonization patterns of these fly species. Further exploration of host and microbial mechanisms that facilitate and govern microbiome assembly are needed for realization of application in SIT programs.

## Methods

### Fly sources and rearing conditions

Insectary sources of melon fly and medfly were obtained from colonies maintained at the USDA-ARS, United States Daniel K. Inouye Pacific Basin Agricultural Reach Center in Hilo, Hawai’I, USA. Colony production followed standard rearing procedures and diets. Larval diet recipes are provided as supporting information (Supplemental Table 5). These colonies have been maintained for 20–30 years (approximately 200 to 400 generations). Wild melon fly pupae were obtained from papaya grown in Kea’au, Hawai’I, USA. Infested papayas were brought to the laboratory where they were maintained in bins on a tray suspended above a vermiculite pupation substrate to contain emerging larvae. Infested papaya fruits were maintained in an open-air, semi-enclosed room, with the following environmental conditions: 22–27 °C, 65–70% relative humidity, and ambient light (~ 12:12). Pupae were collected from the substrate in 3-day windows and maintained in cups until onset of adult emergence.

Flies were reared as cohorts in ~ 20 cm^3^ wood and plexiglass cages under ambient conditions in an open-air room described above. For both laboratory and wild populations, adults were initially provided only a water source for the first 48 h before transferring to a 3:1 ratio of sucrose with USB enzymatic yeast hydrolysate (United States Biochemical, Cleveland, OH, USA). Adults were provided 3:1 sucrose:yeast diet and water throughout sample collection, with both being replaced weekly. No efforts to provide sterile food, water, or rearing containment were made for this study. All experiments took place within a two-week window and collections were performed simultaneously, with insects consuming the same bulk diet source.

### Sample collection

We selected seven timepoints to analyze fly microbiota: pupae (~ 2d pre-eclosion), freshly (< 6 h) eclosed adults (henceforth teneral), 24 h, 48 h, 1 week, 2 weeks, and 3 weeks post adult eclosion for each fly species and combination. For culture-based analysis, both males and females were collected at each timepoint for analysis (n > 12). Only male samples were collected for DNA-based analysis for all but the three-week timepoint (n = 5–8). Flies were randomly selected from the groups and anesthetized on ice for 20 min. Samples were surface sterilized in ice-cold 10% bleach (0.8% sodium hypochlorite) for 1 min, followed by two rinses in autoclaved distilled water. In both analyses, individual flies served as our unit of replication.

Like what other researchers have noted^29^, in our preliminary dissections we observed that teneral adults have delicate digestive systems and that the fragility varied between species. To ensure that we captured total gut microbial titer, we used the whole fly. We later validated that the whole body did not interfere with 16S titer data using qPCR (Supplemental Fig. 1).

### Culture-dependent microbial titer analysis

After surface sterilization, individual flies or fly gut tissues were homogenized in 1 mL of phosphate buffered saline (PBS, pH 7.4) in a 1.7 mL microcentrifuge tube. Samples were plated on LB medium using an Eddy Jet2W spiral plater (IUL S.A. Barcelona, ES). Pupae, teneral, and 24 h-old fly samples were not diluted prior to plating, 48 h were diluted 1:10, and all others diluted 1:100. Plates were incubated at 27 °C for 5–7 days prior to counting. Colony forming units (CFUs) were enumerated using a ProtoCOL3 colony counter (Synbiosis, Frederick, MD, USA).

### DNA extraction and microbiome analysis

Following processing, samples were snap frozen in liquid nitrogen and stored at − 80 °C until DNA extraction. DNA was extracted using a ZymoBIOMICS DNA kit (Zymo, Irvine, CA, USA) following manufacturer recommendations. Extraction plates included ZymoBIOMICS mock community standard and empty control wells as positive and negative controls, respectively. Extracted DNA was quantified using a Qubit dsDNA HS kit (Invitrogen, Waltham, MA, USA).

qPCR was performed using a CFX384 Touch Real-Time PCR thermocycler (Bio-Rad Laboratories; Hercules, CA, USA). We used the primers 1369F (CGGTGAATACGTTCYCGG) and 1492R (GGWTACCTTGTTACGACTT) to amplify a portion of the 16S SSU rRNA^68^. Reactions were conducted using Luna Universal qPCR Master Mix (New England Biolabs, Ipswich, MA, USA) with 5µL reactions composed of 2× master mix, 0.2 µM of each primer, and variable concentrations of template DNA. Conditions were as follows: 95 °C for 1 min, followed by 40 cycles of 95 °C 30 s and 55 °C for 30 s. Standard curves were constructed using serial dilutions of a 192 bp gBlocks double-stranded Gene Fragment (Integrated DNA Technologies, Coralville, IA, USA) synthesized from a partial *E. coli* DH5α 16S sequence with the 1369F-1492R priming sites (Supplemental Information 1).

16S-rRNA metabarcoding was performed using the dual-indexed barcoding strategy described in Kozich et al.^69^. The V4 subregion of the 16S SSU rRNA was amplified using 515F (GTGCCAGCMGCCGCGGTAA) and 806R (GGACTACHVGGGTWTCTAAT), with primers designed to include Illumina index sequences and sequence barcodes. Reactions were performed in 25 µL volumes with Q5 Hot Start High-Fidelity Polymerase (New England Biolabs), 0.2 µM of each primer, and variable inputs of template DNA. Reaction conditions were as follows: 98 °C 30 s, 30 cycles of 98 °C 30 s, 50 °C 30 s, and 72 °C 2 min, and a final extension step at 72 °C for 10 min. Teneral and 24 h-post eclosion samples that exhibited low 16S rRNA abundance (see qPCR/culturing results) were amplified in triplicate prior to pooling to obtain quantifiable DNA. PCR products were purified using size-selection magnetic bead cleanup and products were quantified using Quant-IT PicoGreen (Invitrogen, Waltham, MA, USA) using a Spectramax M2 (Molecular Devices, San Jose, CA, USA). Samples were pooled in approximately equimolar concentrations. Amplicon pools were sequenced at the ASGPB Genomics Core at the University of Hawai’i at Manoa using Illumina MiSeq V3 600 base-pair chemistry (Illumina, San Diego, CA, USA) to generate 300 base-pair paired-end reads.

### Processing of sequence data

Illumina 16S reads were processed with the ‘DADA2’ (v. 1.24) pipeline to obtain amplicon sequence variants (ASVs)^70^ implemented in R. Steps included filtering, dereplication, inference of sequence variants, mergers of paired end reads, and chimera detection and removal. Taxonomy assignments were performed using ‘DECIPHER’ (v. 2.24.0)^71^ with a trained version of the Ribosomal Database Project (RDP) reference database (v 18). ASVs assigned to chloroplast, mitochondria, or those which were unclassified at the domain level were removed from the dataset prior to statistical analyses. The taxonomic classifier IDTAXA implemented in DECIPHER is more conservative than other classifiers, and returns less false positives than other classifiers^72^. DECIPHER returned a large number of unclassified sequences at the genus level (see Results; Fig. 5), so we also performed classifications of ASVs with the RDP naïve Baysian classifier with the v18 database^73^ and report those results alongside the DECIPHER classifications.

We included a negative kit control and positive mock communities in our extractions and PCRs as described above and sequenced them alongside our samples. The negative control yielded no sequences at the end of the pipeline. The positive control approximated the community composition of the mock community (Supplemental Fig. 6).

### Statistical analyses

Statistical analyses were performed using R 4.2.1 in RStudio^74,75^. Culture titer data and 16S-rRNA qPCR data were analyzed using an ANOVA with log_10_ transformed response variables with pairwise comparisons between timepoints using an FDR correction. Separate analyses were performed with the different species/sources.

Microbiome data were analyzed using the ‘VEGAN’ package^76^ after processing with DADA2. Each species was analyzed separately for each timepoint, unless otherwise specified in the results. Specimens were rarefied to 1175 sequences at this point (Supplemental Fig. 2). Alpha-diversity metrics of ASV richness, Shannon, and 1/Simpson metrics were computed in VEGAN and analyzed using a Kruskal–Wallis test with pairwise comparisons being performed with Dunn tests implemented in the package ‘FSA’^77^. ASV composition and membership were analyzed using Bray–Curtis and Jaccard distances, respectively. Distances were visualized and analyzed using non-metric multidimensional scaling (NMDS) and vegan::adonis. Pairwise multivariate comparisons were performed using the package ‘pairwiseAdonis’^78^. Differences in individual ASV relative abundances were analyzed using Kruskal–Wallis tests. Heatmaps were produced by using a Z-transformation of ASV relative abundances within a sample and illustrated using ggplot2.

### Supplementary Information

Supplementary Information.

## Data Availability

Raw sequence reads have been deposited to NCBI Sequence Read Archive under the accession PRJNA957317. Other data and R analysis has been deposited at the USDA NAL Ag Data Commons at 10.15482/USDA.ADC/1529269.
