# Author Correction: Gut bacterial population and community dynamics following adult emergence in pest tephritid fruit flies

**DOI:** 10.1038/s41598-023-43742-2

**Published:** 2023-10-03

**Authors:** Charles J. Mason, Jean Auth, Scott M. Geib

**Affiliations:** Tropical Pest Genetics and Molecular Biology Research Unit, Daniel K Inouye U.S. Pacific Basin Agricultural Research Center, Agricultural Research Service, USDA, 64 Nowelo Street, Hilo, HI 96720 USA

Correction to: *Scientific Reports* https://doi.org/10.1038/s41598-023-40562-2, Published online 22 August 2023

The original version of this Article contained an error in the Data availability section where the link to the dataset was incorrect.

“Raw sequence reads have been deposited to NCBI Sequence Read Archive under the accession PRJNA95317. Other data and R analysis has been deposited at the USDA NAL Ag Data Commons at https://doi.org/10.15482/USDA.ADC/1529269.”

now reads:

“Raw sequence reads have been deposited to NCBI Sequence Read Archive under the accession PRJNA957317. Other data and R analysis has been deposited at the USDA NAL Ag Data Commons at https://doi.org/10.15482/USDA.ADC/1529269.”

The original Article has been corrected.

