# Supplementary material for: Gut bacterial population and community dynamics following adult emergence in pest tephritid fruit flies
Source: Sci Rep. 2023 Aug 22;13:13723. doi: 10.1038/s41598-023-40562-2 (PMC10444893; doi:10.1038/s41598-023-40562-2)
Supplement: Supplementary file 1 — Supplementary Information. [file 41598_2023_40562_MOESM1_ESM.docx]

| Supplemental Table 1: Changes in fly gut microbial alpha diversity metrics at different timepoints. Numbers represent mean (interquartile range). Letters represent significant differences determined with a Dunn Kruskal-Wallis comparison using a Benjamini-Hochberg p-value adjustment. | | | | | | | | |
| --- | --- | --- | --- | --- | --- | --- | --- | --- |
| Species & Metric: | | Timepoint | | | | | |  |
| Melon Fly (Papaya) | | Pupa | Teneral | 24H | 48H | 1W | 2W | p-value  (Kruskal-Wallis) |
|  | Shannon: | 2.708 (0.739)a | 2.027 (1.354)ab | 1.713 (1.074)ab | 1.419 (0.541)b | 1.694 (1.397)ab | 2.311 (0.758)ab | **0.029** |
|  | Inverse Simpson | 1.136 (0.08)b | 1.325 (0.393)ab | 2.516 (0.687)ab | 2.075 (0.387)a | 1.628 (0.404)a | 1.503 (0.216)a | **0.002** |
|  | Obs. OTUs | 46.9 (43.5) | 23.5 (15) | 38 (43.3) | 20.6 (21.5) | 27.6 (42.5) | 42 (15.5) | 0.199 |
| Melon Fly (lab) | |  |  |  |  |  |  |  |
|  | Shannon: | 1.693 (0.842)ab |  | 1.088 (0.398)b | 1.022 (0.648)b | 2.119 (1.833)ab | 2.572 (0.515)a | **0.021** |
|  | Inverse Simpson | 1.552 (0.34)ab |  | 9.161 (0.408)b | 2.691 (1.306)ab | 1.66 (0.971)ab | 1.227 (0.132)a | **0.023** |
|  | Obs. OTUs | 27.8 (18)ab |  | 9 (1)ab | 14.7 (9.5)b | 44.2 (48.8)ab | 51.9 (9)a | **0.039** |
| Medfly (lab) | |  |  |  |  |  |  |  |
|  | Shannon: | 1.516 (0.092)ab | 2.616 (0.396)b | 1.657 (0.848)ab | 1.427 (0.072)ab | 0.935 (0.228)b | 1.323 (0.458)b | **0.002** |
|  | Inverse Simpson | 1.498 (0.102)ab | 1.164 (0.029)b | 1.852 (0.422)ab | 1.737 (0.094)ab | 2.162 (0.392)a | 1.837 (0.651)a | **0.005** |
|  | Obs. OTUs | 22.4 (8)abc | 45.2 (11)b | 33 (6)bc | 21.4 (3)abc | 10.7 (6.8)a | 12 (7.8)a | **0.001** |
| Oriental Fruit Fly (lab) | |  |  |  |  |  |  |  |
|  | Shannon: | 1.104 (0.927) | 2.09 (0.504) | 1.755 (0.037) |  | 1.794 (0.213) | 1.64 (0.415) | 0.152 |
|  | Inverse Simpson | 6.202 (2.373) | 1.544 (0.375) | 4.631 (0.127) |  | 1.41 (0.154) | 1.692 (0.528) | 0.386 |
|  | Obs. OTUs | 16.2 (7) | 32 (9) | 35.2 (25) |  | 22.3 (5.8) | 24.9 (10) | 0.178 |

| Supplemental Table 2: Responses of individual OTUs at different timepoints for melon fly from papaya. Values represent relative abundances. First line in taxonomy is form DECIPHER with second being RDP Naïve Bayesian classifier (with bootstrap values). | | | | | | | | |
| --- | --- | --- | --- | --- | --- | --- | --- | --- |
| ASV | p-value | Timepoint: | | | | | | Taxonomy |
|  |  | Pupa | Teneral | 24H | 48H | 1W | 2W |  |
| ASV17 | 0.014 | 0.034 | 0.165 | 0 | 0.001 | 0 | 0 | Unclassified Enterobacteriaceae \| Unclassified Enterobacteriaceae-19% |
| ASV27 | 0.009 | 0.014 | 0.069 | 0 | 0 | 0 | 0 | Unclassified Enterobacterales \| Unclassified Enterobacteriaceae-12% |
| ASV514 | 0.021 | 0.001 | 0 | 0 | 0 | 0 | 0 | Leuconostoc \| Leuconostoc-100% |
| ASV7 | 0.029 | 0.008 | 0.001 | 0 | 0.000 | 0.001 | 0 | Acinetobacter \| Acinetobacter-100% |
| ASV84 | 0.021 | 0.028 | 0.029 | 0.000 | 0 | 0.002 | 0 | Unclassified Enterobacteriaceae \| Escherichia/Shigella-95% |
| ASV89 | 0.021 | 0.023 | 0.001 | 0 | 0 | 0 | 0 | Unclassified Weeksellaceae \| Faecalibacter-52% |
|  |  |  |  |  |  |  |  |  |
| Collective Change (Sum of Relative Abundance) | |  |  |  |  |  |  |  |
|  |  | 0.108 | 0.264 | 0.000 | 0.001 | 0.002 | 0.000 |  |

| Supplemental Table 3: Responses of individual OTUs at different timepoints for melon fly from insectary. Values represent relative abundances. First line in taxonomy is form DECIPHER with second being RDP Naïve Bayesian classifier (with bootstrap values). | | | | | | | |
| --- | --- | --- | --- | --- | --- | --- | --- |
| ASV | p-value | Timepoint: | | | | | Taxonomy |
|  |  | Pupa | 24H | 48H | 1W | 2W |  |
| ASV1 | 0.033 | 0.028 | 0.001 | 0.107 | 0.237 | 0.229 | Unclassified Enterobacteriaceae \| Klebsiella-98% |
| ASV107 | 0.020 | 0 | 0 | 0 | 0.001 | 0.005 | Unclassified Enterobacteriaceae \| Klebsiella-56% |
| ASV11 | 0.015 | 0.014 | 0 | 0.002 | 0 | 0 | Staphylococcus \| Staphylococcus-73% |
| ASV121 | 0.023 | 0 | 0 | 0 | 0.002 | 0.005 | Unclassified Enterobacteriaceae \| Klebsiella-71% |
| ASV131 | 0.020 | 0 | 0 | 0 | 0.002 | 0.004 | Unclassified Enterobacteriaceae \| Klebsiella-71% |
| ASV19 | 0.030 | 0.002 | 0 | 0 | 0 | 0 | Brucella \| Brucella-97% |
| ASV22 | 0.016 | 0.006 | 0 | 0 | 0 | 0 | Pseudochrobactrum \| Brucella-84% |
| ASV27 | 0.030 | 0.005 | 0 | 0 | 0 | 0 | Unclassified Enterobacterales \| Unclassified Enterobacteriaceae-12% |
| ASV42 | 0.020 | 0 | 0 | 0 | 0.002 | 0.013 | Unclassified Enterobacteriaceae \| Klebsiella-74% |
| ASV43 | 0.020 | 0 | 0 | 0 | 0.003 | 0.013 | Unclassified Enterobacteriaceae \| Klebsiella-73% |
| ASV45 | 0.021 | 0 | 0 | 0 | 0.005 | 0.009 | Providencia \| Providencia-99% |
| ASV47 | 0.020 | 0 | 0 | 0 | 0.003 | 0.011 | Unclassified Enterobacteriaceae \| Klebsiella-79% |
| ASV49 | 0.020 | 0 | 0 | 0 | 0.003 | 0.011 | Unclassified Enterobacterales \| Unclassified Enterobacteriaceae-39% |
| ASV5 | 0.002 | 0.025 | 0 | 0 | 0 | 0 | Unclassified Enterobacteriaceae \| Raoultella-60% |
| ASV52 | 0.022 | 0 | 0 | 0 | 0.007 | 0.008 | Providencia \| Providencia-99% |
| ASV54 | 0.034 | 0 | 0 | 0.001 | 0.005 | 0.010 | Providencia \| Providencia-100% |
| ASV56 | 0.020 | 0 | 0 | 0 | 0.003 | 0.011 | Unclassified Enterobacteriaceae \| Unclassified Enterobacteriaceae-2% |
| ASV59 | 0.020 | 0 | 0 | 0 | 0.003 | 0.011 | Unclassified Enterobacteriaceae \| Klebsiella-68% |
| ASV6 | 0.020 | 0.047 | 0.000 | 0.005 | 0 | 0 | Staphylococcus \| Staphylococcus-99% |
| ASV61 | 0.020 | 0 | 0 | 0 | 0.003 | 0.009 | Unclassified Enterobacteriaceae \| Klebsiella-80% |
| ASV63 | 0.022 | 0 | 0 | 0 | 0.006 | 0.007 | Providencia \| Providencia-97% |
| ASV66 | 0.022 | 0 | 0 | 0 | 0.006 | 0.006 | Providencia \| Providencia-99% |
| ASV67 | 0.021 | 0 | 0 | 0 | 0.003 | 0.007 | Unclassified Enterobacteriaceae \| Klebsiella-64% |
| ASV71 | 0.021 | 0 | 0 | 0 | 0.005 | 0.006 | Providencia \| Providencia-99% |
| ASV73 | 0.021 | 0 | 0 | 0 | 0.003 | 0.007 | Unclassified Enterobacteriaceae \| Klebsiella-55% |
| ASV79 | 0.025 | 0 | 0 | 0 | 0.007 | 0.006 | Providencia \| Providencia-100% |
| ASV81 | 0.021 | 0 | 0 | 0 | 0.002 | 0.007 | Unclassified Enterobacteriaceae \| Unclassified Enterobacteriaceae-20% |
| ASV99 | 0.020 | 0 | 0 | 0 | 0.002 | 0.006 | Unclassified Enterobacterales \| Klebsiella-71% |
|  |  |  |  |  |  |  |  |
| Collective Change (Sum of Relative Abundance) | |  |  |  |  |  |  |
|  |  | 0.128 | 0.001 | 0.115 | 0.312 | 0.399 |  |

| Supplemental Table 4: Responses of individual OTUs at different timepoints for medfly. Values represent relative abundances. First line in taxonomy is form DECIPHER with second being RDP Naïve Bayesian classifier (with bootstrap values). | | | | | | | | |
| --- | --- | --- | --- | --- | --- | --- | --- | --- |
| ASV | p-value | Timepoint: | | | | | | Taxonomy |
|  |  | Pupa | Teneral | 24H | 48H | 1W | 2W |  |
| ASV1 | 0.042 | 0.001 | 0.091 | 0.047 | 0.249 | 0.393 | 0.191 | Unclassified Enterobacteriaceae \| Klebsiella-98% |
| ASV101 | 0.009 | 0 | 0 | 0.001 | 0.008 | 0 | 0 | Sphingobacterium \| Sphingobacterium-100% |
| ASV106 | 0.007 | 0.016 | 0.005 | 0.002 | 0.000 | 0 | 0 | Brachybacterium \| Brachybacterium-100% |
| ASV11 | 0.001 | 0.301 | 0.041 | 0.010 | 0.001 | 0 | 0 | Staphylococcus \| Staphylococcus-73% |
| ASV12 | 0.002 | 0.000 | 0.027 | 0.439 | 0.005 | 0 | 0 | Pseudomonas \| Pseudomonas-92% |
| ASV120 | 0.001 | 0.008 | 0 | 0 | 0 | 0 | 0 | Staphylococcus \| Staphylococcus-97% |
| ASV122 | 0.003 | 0 | 0.001 | 0.012 | 0.002 | 0 | 0 | Unclassified Comamonadaceae \| Delftia-89% |
| ASV126 | 0.003 | 0 | 0.043 | 0.004 | 0.004 | 0 | 0 | Unclassified Comamonadaceae \| Comamonas-92% |
| ASV136 | 0.004 | 0.010 | 0.003 | 0.000 | 0 | 0 | 0 | Unclassified Micrococcaceae \| Kocuria-65% |
| ASV14 | 0.002 | 0 | 0 | 0.132 | 0.008 | 0 | 0 | Pseudomonas \| Pseudomonas-97% |
| ASV140 | 0.003 | 0.001 | 0.019 | 0.001 | 0 | 0 | 0 | Pseudomonas \| Pseudomonas-100% |
| ASV152 | 0.002 | 0.009 | 0.001 | 0 | 0 | 0 | 0 | Brachybacterium \| Brachybacterium-100% |
| ASV18 | 0.005 | 0 | 0 | 0.043 | 0.018 | 0 | 0 | Pseudomonas \| Pseudomonas-91% |
| ASV209 | 0.001 | 0.006 | 0 | 0 | 0 | 0 | 0 | Unclassified Staphylococcaceae \| Unclassified Staphylococcaceae-27% |
| ASV215 | 0.002 | 0 | 0 | 0.004 | 0.002 | 0 | 0 | Variovorax \| Variovorax-99% |
| ASV224 | 0.013 | 0.003 | 0 | 0.001 | 0 | 0 | 0 | Unclassified Micrococcaceae \| Nesterenkonia-72% |
| ASV229 | 0.004 | 0.004 | 0.000 | 0.000 | 0 | 0 | 0 | Unclassified Staphylococcaceae \| Unclassified Staphylococcaceae-45% |
| ASV23 | 0.005 | 0 | 0 | 0.019 | 0.052 | 0 | 0 | Acinetobacter \| Acinetobacter-100% |
| ASV235 | 0.004 | 0 | 0 | 0.003 | 0.002 | 0 | 0 | Caulobacter \| Caulobacter-100% |
| ASV247 | 0.001 | 0.003 | 0 | 0 | 0 | 0 | 0 | Brachybacterium \| Brachybacterium-100% |
| ASV277 | 0.003 | 0 | 0.009 | 0.001 | 0.000 | 0 | 0 | Unclassified Rhizobiaceae \| Agrobacterium-100% |
| ASV3 | 0.004 | 0.000 | 0 | 0.010 | 0.010 | 0.057 | 0.016 | Morganella \| Morganella-99% |
| ASV306 | 0.009 | 0 | 0.010 | 0.001 | 0 | 0 | 0 | Unclassified Pseudomonadaceae \| Azotobacter-72% |
| ASV31 | 0.002 | 0.071 | 0.018 | 0.003 | 0.000 | 0 | 0 | Brevibacterium \| Brevibacterium-100% |
| ASV34 | 0.003 | 0.043 | 0.006 | 0.004 | 0 | 0 | 0 | Staphylococcus \| Staphylococcus-99% |
| ASV344 | 0.024 | 0.001 | 0.001 | 0 | 0 | 0 | 0 | Dietzia \| Dietzia-97% |
| ASV36 | 0.014 | 0 | 0 | 0 | 0 | 0.002 | 0 | Unclassified Enterobacteriaceae \| Klebsiella-78% |
| ASV4 | 0.020 | 0.001 | 0.065 | 0.006 | 0.024 | 0 | 0.366 | Unclassified Enterobacteriaceae \| Klebsiella-97% |
| ASV41 | 0.006 | 0.037 | 0.006 | 0.003 | 0.000 | 0 | 0 | Brevibacterium \| Brevibacterium-100% |
| ASV454 | 0.013 | 0 | 0.008 | 0.001 | 0 | 0 | 0 | Pseudomonas \| Pseudomonas-99% |
| ASV51 | 0.003 | 0 | 0 | 0 | 0 | 0 | 0.047 | Providencia \| Providencia-96% |
| ASV558 | 0.027 | 0.001 | 0 | 0 | 0 | 0 | 0 | Brevibacterium \| Brevibacterium-100% |
| ASV6 | 0.001 | 0.469 | 0.177 | 0.073 | 0.003 | 0 | 0 | Staphylococcus \| Staphylococcus-99% |
| ASV605 | 0.027 | 0 | 0.004 | 0 | 0 | 0 | 0 | Massilia \| Massilia-100% |
| ASV623 | 0.027 | 0 | 0.002 | 0 | 0 | 0 | 0 | Methylobacterium \| Methylobacterium-100% |
| ASV7 | 0.004 | 0 | 0.004 | 0.069 | 0.305 | 0 | 0 | Acinetobacter \| Acinetobacter-100% |
| ASV74 | 0.003 | 0 | 0 | 0 | 0 | 0 | 0.038 | Providencia \| Providencia-99% |
| ASV9 | 0.003 | 0 | 0.273 | 0.014 | 0.001 | 0.278 | 0.047 | Unclassified Enterobacterales \| Serratia-60% |
| ASV98 | 0.011 | 0 | 0 | 0.000 | 0.006 | 0 | 0 | Unclassified Enterobacterales \| Pantoea-97% |
| Collective Change (Sum of Relative Abundance) | |  |  |  |  |  |  |  |
|  |  | 0.985 | 0.813 | 0.901 | 0.701 | 0.730 | 0.705 |  |

| Supplemental Table 5: Diet formulations for USDA PBARC fruit fly colonies. | | | |
| --- | --- | --- | --- |
| Ingredient (in g): | Medfly | Melon Fly | Oriental Fruit Fly |
| Wheat Mill Feed | 2322 (27.5 %) | 2460 (31.4%) | 2377.5 (28.7%) |
| Granulated Sugar | 1075.5 (12.7%) | 580.5 (7.4%) | 1077 (13.0%) |
| Torula Yeast | 300 (3.6%) | 280.5 (3.6%) | 300 (3.6%) |
| Citric Acid | 204 (2.4%) | -- | -- |
| Nipagen | 18.3 (0.23%) | 9 (0.11%) | 10.1 (0.12%) |
| Sodium Benzoate | 18.3 (0.23% | 9 (0.11%) | 8.4 (0.10%) |
| Water (in mL) | 4500 (53.3%) | 4500 (57.4%) | 4500 (54.4%) |

CTAGTAATCGTGGATCAGAATGCCACGGTGAATACGTTCCCGGGCCTTGTACACACCGCCCGTCACACCATGGGAGTGGGTTGCAAAAGAAGTAGGTAGCTTAACCTTCGGGAGGGCGCTTACCACTTTGTGATTCATGACTGGGGTGAAGTCGTAACAAGGTAACCGTAGGGGAACCTGCGGTTGGATCA

**Supplemental Material 1:** Partial 16S SSU rRNA sequence of *E.coli* dh5α used to synthesize standard curve for qPCR. Green and yellow highlighted regions correspond to the location of the forward (1369F) and reverse (1492R) primer sequences, respectively.

**Supplemental Figure 1**: Comparisons between different fly species at pupal stages (A), 24h post-eclosion (B), one-week post-eclosion (B), and two-week post-eclosion (C). Left column NMD scores were calculated with Bray-Curtis distances while the right column scores were calculated with Jaccard distances.


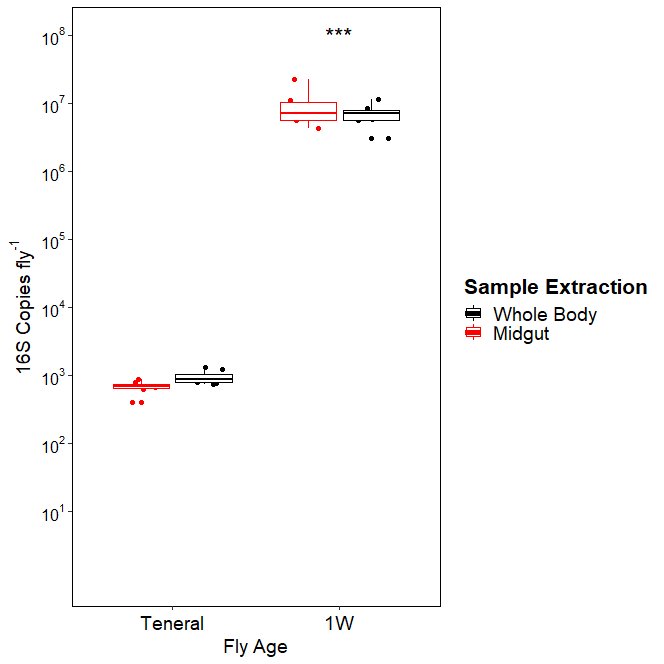


**Supporting Figure 1**: 16S rRNA copy numbers of laboratory-reared melon flies from DNA extracted from the whole body vs. a dissected midgut. (*** = p < 0.001).


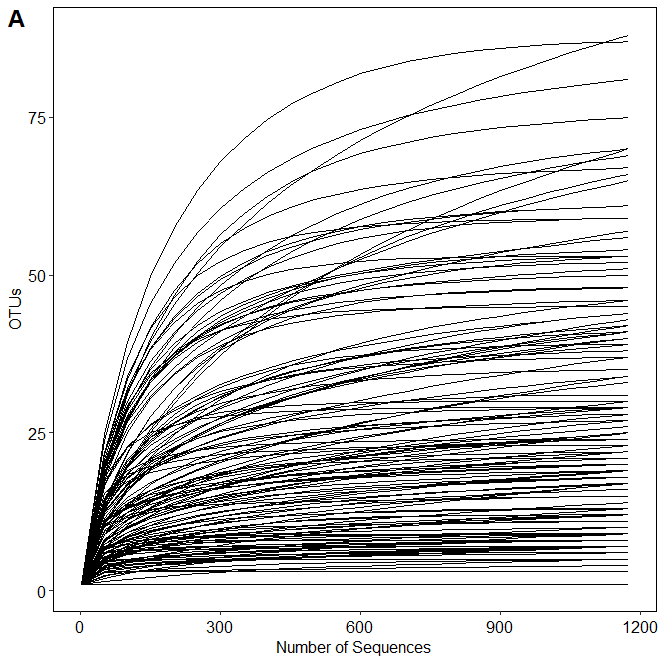


**Supplemental Figure 2**: Sample accumulation curves of rarefied samples.


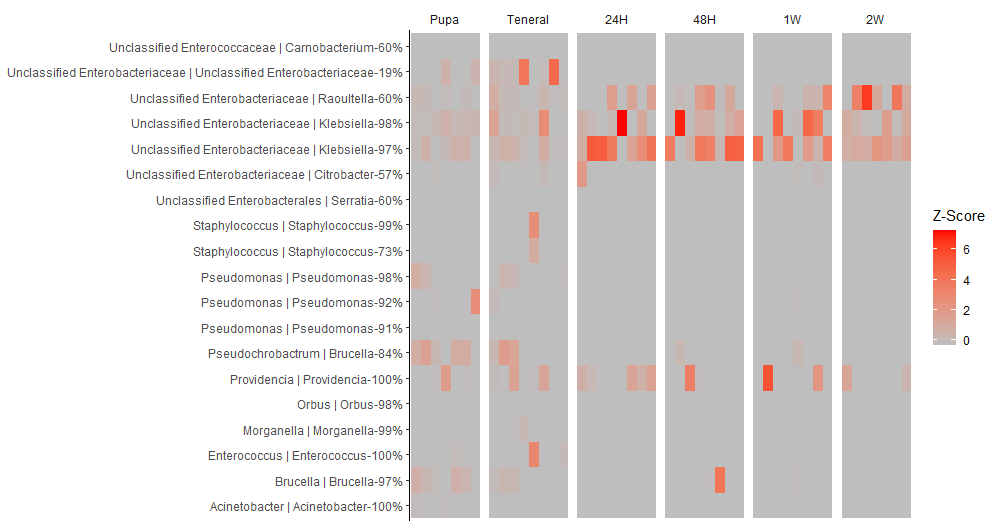


**Supplemental Figure 3**: Heatmap of ASVs associated with melon fly pupae collected from papaya. Both taxonomies determined from DECIPHER and RDP are included (left, and right, respectively), with the RDP bootstrap value at the genus level included.


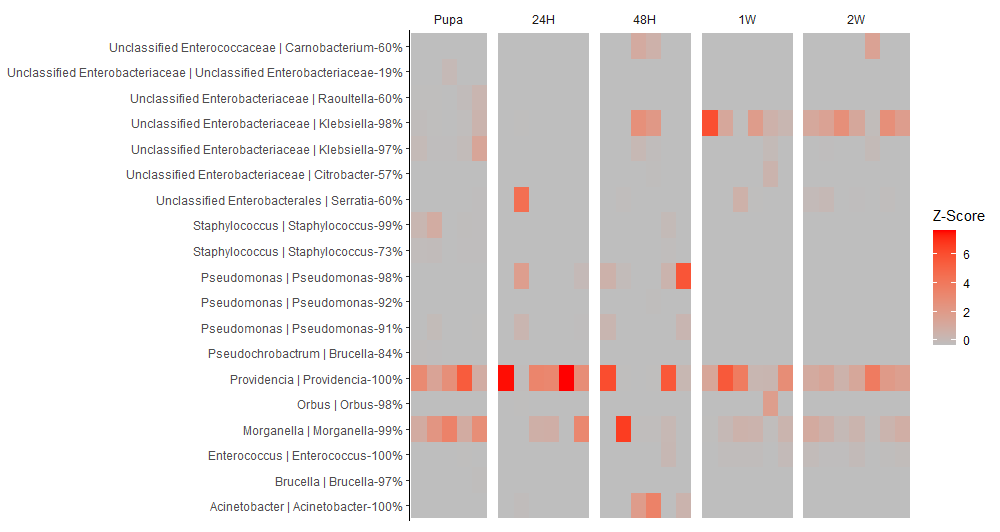


**Supplemental Figure 4**: Heatmap of ASVs associated with melon fly collected from the laboratory. Both taxonomies determined from DECIPHER and RDP are included (left, and right, respectively), with the RDP bootstrap value at the genus level included.


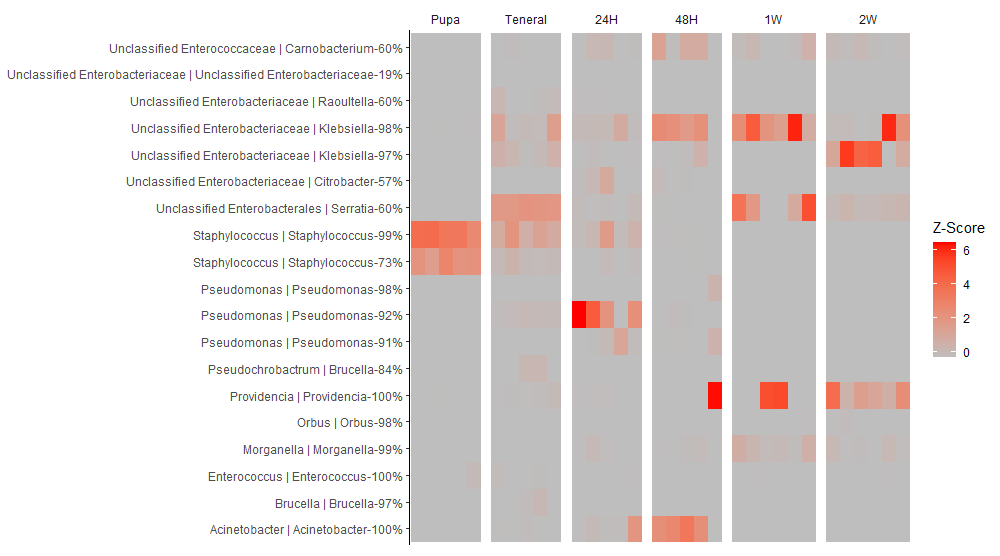


**Supplemental Figure 5**: Heatmap of ASVs associated with medfly from the laboratory. Both taxonomies determined from DECIPHER and RDP are included (left, and right, respectively), with the RDP bootstrap value at the genus level included.


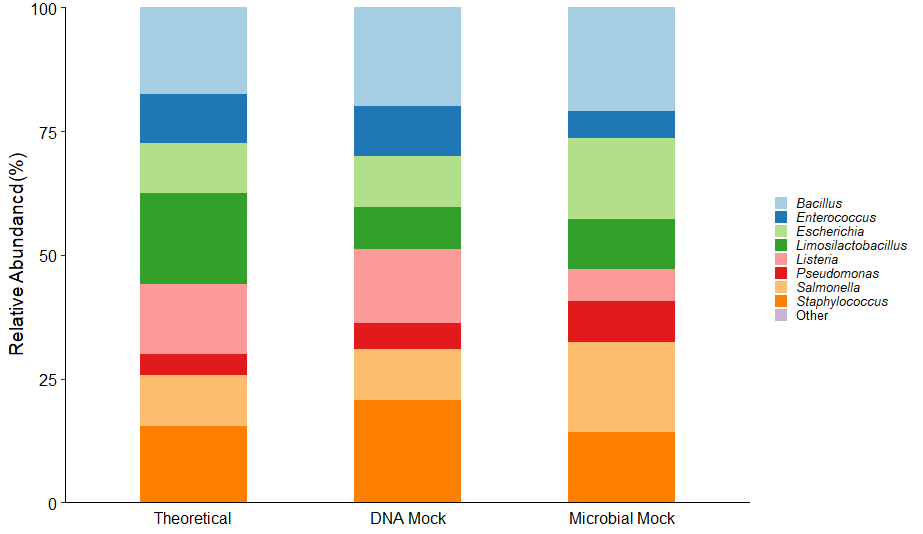


**Supplementary Figure 6**: Performance of mock community in DNA extraction and sequencing pipeline as displayed by relative abundance of classified ASVs (%). For the DNA mock community (Zymo; only PCR performed), the eight anticipated isolates were split across eleven ASVs, with nine comprising >99% of the reads. For the microbial mock community (Zymo; all extraction and PCR performed), the eight isolates were split into ten ASVs, eight of which comprised >99% of the reads.
